# Sublethal effects of imidacloprid on the fitness of two species of wheat aphids, *Schizaphis graminum* (R.) and *Rhopalosiphum padi* (L.)

**DOI:** 10.1371/journal.pone.0294877

**Published:** 2023-11-27

**Authors:** Xiang Ji, Yu-Tai Jiang, Tian-Xin Guo, Pei Zhang, Xin-an Li, Fan-Bin Kong, Bai-Zhong Zhang

**Affiliations:** 1 Hebi Institute of Engineering and Technology, Henan Polytechnic University, Hebi, P.R. China; 2 College of Resources and Environment, Henan Engineering Research Center of Biological Pesticide & Fertilizer Development and Synergistic Application, Henan Institute of Science and Technology, Xinxiang, P.R. China; 3 Department of Entomology, China Agricultural University, Beijing, P.R. China; Institut Sophia Agrobiotech, FRANCE

## Abstract

Imidacloprid is a neonicotinoid insecticide that efficiently controls piercing-sucking mouthparts pests. However, the impact of low lethal concentration of imidacloprid on key demographic parameters of wheat aphids, *Schizaphis graminum* (R.) and *Rhopalosiphum padi* (L.) has been scarcely studied. In this study, we used the age stage, two-sex life table approach to investigate the sublethal effects of imidacloprid on the biological traits of *S*. *graminum* and *R*. *padi*. Bioassays showed that imidacloprid possesses high toxicity to adult *S*. *graminum* and *R*. *padi*, with LC_50_ of 3.59 and 13.78 mg L^−1^ following 24 h exposure. A low lethal concentration of imidacloprid (LC_25_) significantly decreased adult longevity and total longevity of progeny generation aphids (F_1_) of *S*. *graminum*. Nevertheless, imidacloprid (LC_25_) had no significant effects on the fecundity and longevity of directly exposed parental parental *S*. *graminum* and *R*. *padi* (F_0_). Our results showed that the low lethal concentration of imidacloprid affected the demographic parameters that ultimately impact on the population of *S*. *graminum*. This study provides detailed information about the overall effects of imidacloprid on *S*. *graminum* and *R*. *padi* that might help to manage these two key pests.

## Introduction

Wheat aphids are the main agricultural pests in grain-growing areas of the world [1, 2]. *Schizaphis graminum* (R.)(Sg)and *Rhopalosiphum padi* (Rp)(L.) are important pests in wheat fields in China. They can cause severe damage to wheat crops, and they can also spread barley yellow dwarf virus, resulting in a large yield reduction and serious economic losses [3–6]. At present, chemical insecticides are the main method of controlling wheat aphids in the field, but long-term unscientific use of insecticides can lead to the development of wheat aphid resistance [7, 8]. In addition, insecticides gradually degrade to low lethal or sublethal concentrations after field application, resulting in sublethal effects on pests [9–12].

Sublethal effects are defined as impacts on the physiology and/or behaviour of individuals that survived exposure to insecticides at low lethal or sublethal concentrations [13]. Insecticides may have sublethal effects on insect physiology and behaviour [14], such as insect survival [15, 16], developmental duration [17–20], and fecundity [21–23].

Santos et al. [15] found that females of the Neotropical brown stink bug *Euschistus heros* had reduced rates of survival but higher fecundity and fertility rates under sublethal imidacloprid conditions. Chen et al. [18] found that exposure of *Aphis gossypii* Glover to imidacloprid at a low lethal concentration (LC_25_) significantly increased the duration of their preadult stage and total preoviposition period as well as their mean generation time. Yuan et al. [22] found that the adults of cotton aphids.

*(Aphis gossypii* Glover) treated with a low lethal concentration (LC_10_) and a lethal concentration (LC_40_) of cycloxaprid had lower adult fecundity and net productive rates than controls.

Therefore, evaluating the sublethal effects of insecticides on pests provides better understanding of the response of pests to insecticides and has positive practical importance for guiding rational insecticide use in the field [9, 24, 25].

Imidacloprid acts on the acetylcholinesterase receptor in the nervous system of pests, interfering with the normal transmission of signals in their central nervous system and causing accumulation of high levels of acetylcholine; this causes a state of continuous excitation until paralysis and death. Therefore, it has high insecticidal activity and is characterized by high efficiency, low toxicity and a wide insecticidal spectrum [26–28]. Imidacloprid has a variety of effects on pests. It can be quickly absorbed by plants after application, so it has high control effects on piercing-sucking pests [29, 30], and it has been widely used to control wheat aphids in China.

Low or sublethal concentrations of insecticide typically occur due to field degradation and plant growth after the initial insecticide application [31]. Therefore, under field conditions, wheat aphids may be exposed to low lethal or sublethal concentrations of insecticides and exhibit sublethal effects on behaviour and physiology, with potential transgenerational transmission to the offspring of these surviving individuals [13, 32]. Xiao et al. [25] found that *Sitobion avenae* and *R*. *padi* have different responses exposure to pirimicarb. He et al. [33] found that sublethal concentrations of imidacloprid and bifenthrin significantly reduced phloem feeding, honeydew excretion and reproductive ability in *Bemisia tabaci*, while sublethal concentrations of chlorpyrifos and carbosulfan did not exert these harmful effects. Cui et al. [34] found that a sublethal concentration of cycloxaprid had a negative impact on the phloem-feeding behaviour and growth rate of *Sitobion avenae* in contact activity and root activity tests of cycloxaprid. Lashkari et al. [35] found that exposure of *Brevicoryne brassicae* to sublethal concentrations of imidacloprid and pymetrozine significantly reduced the intrinsic rate of increase (*r*_*m*_) and the average fecundity of each female aphid and increased the mean generation time.

The construction and analysis of life tables can comprehensively describe population dynamics and help to clarify the sublethal effects of insecticides on pests [36]. Therefore, to evaluate the sublethal effects of imidacloprid on *S*. *graminum* and *R*. *padi*, life tables were constructed and analysed. This study is expected to guide scientific and rational insecticide use in the field and optimize the management of two important wheat aphids.

## Materials and methods

### Insects and insecticides

*S*. *graminum* and *R*. *padi* were fed fresh seedlings of Zhoumai 18 wheat and were reared under laboratory conditions with a temperature of 23–25°C, a relative humidity of 50%-65%, and a photoperiod of 14 h:10 h (L:D). During this period, they were kept from contact with any chemicals. Imidacloprid (95% active ingredient, w/w) was obtained from Dow AgroSciences Inc. (USA).

### Insecticide bioassays

The lethal concentration of imidacloprid for aphids was determined by the aphid-dipping method as described previously [37]. The original imidacloprid (95%) was weighed, and then a certain amount of acetone was added to prepare a 1000 mg/L stock solution. The 5 concentrations (0.4-, 0.8-, 1.6-, 3.2-, and 6.4 mg L^−1^ for *S*. *graminum*; 5-, 10-, 15-, 20-, and 25 mg L^−1^ for *R*. *padi*) tested in the current study were created by dilution with 0.05% (v/v) TX-100 (surfactant) water.

Three repetitions of 25 aphids received treatment with one of these concentrations. Healthy wingless adult aphids raised on wheat seedlings were gently picked up with a writing brush, placed on fresh leaves, and then immersed in the imidacloprid solution for 10 s. After that, the leaves and aphids were removed with filter paper. The aphids were placed into a marked glass tube (1.5 cm in diameter, 7 cm in length) that was sealed with cotton. Surfactant was additionally used as a control treatment. After treatment, the aphids were reared under normal room conditions (temperature of 23–25°C, relative humidity of 50%-65%, photoperiod of 14 h:10 h (L:D). After 24 h, the aphids in each treatment were observed and recorded. The standard for judging the death of aphids is that only one leg moves or no movement is observed [38]. The LC_25_ values of imidacloprid for *S*. *graminum* and *R*. *padi* were estimated using the bioassay results of imidacloprid; these concentrations were used in the experiments to determine the sublethal and transgenerational effects of imidacloprid. The obtained data were regarded as effective if the mortality rate of the control group was less than 5%, and PoloPlus software was used to carry out statistical analysis to determine the LC_25_ and LC_50_ values of imidacloprid on *S*. *graminum* and *R*. *padi*.

### Sublethal and transgenerational effects of imidacloprid on *S*. *graminum* and *R*. *padi*

The sublethal and transgenerational effects of imidacloprid on two wheat aphids were investigated based on the experimental design of the life table. Wheat seedlings (Zhoumai 18) were placed on the bottom of disposable cups (top: 7.5 cm in diameter, bottom: 5.0 cm in diameter, 4.2 cm in height) lined with moist filter paper to maintain the humidity level. The imidacloprid stock solution was diluted to LC_25_ with distilled water containing 0.05% surfactant (Triton X-100). The *S*. *graminum* and *R*. *padi* adults on wheat seedlings were immersed in the LC_25_ of imidacloprid for 10 s. Mortality was calculated at 24 h after treatment, and 40 surviving aphids were collected and reared in plastic dishes containing new wheat seedlings (Zhoumai 18), labelled LC_25__Sg_F_0_ and LC_25__Rp_F_0_. The control group was treated with 0.05% surfactant (Triton X-100) in distilled water and an equal volume of acetone; these groups were labelled CK_Sg_F_0_ and CK_Rp_F_0_. Therefore, four groups (LC_25__Sg_F_0_, CK_Sg_F_0_, LC_25__Rp_F_0_ and CK_Rp_F_0_) were established over 40 repetitions. The adult longevity of the F_0_ generation aphids and the number of new nymphs produced in each replicate were recorded every 12 h until the adults died. After daily counts, F_1_ generation nymphs were removed from the disposable cups.

New nymphs of the F_1_ generation (age<24 h) were selected from each group, and replicates in the F_1_ generation of the treatment and control groups were reared in disposable cups containing new wheat seedlings (Zhoumai 18). Forty repetitions were established for the four groups (CK_Sg_F_1_, LC_25__Sg_F_1_, CK_Rp_F_1_, and LC_25__Rp_F_1_). Thereafter, observations were made every 12 h to record the developmental time in each growth stage of the F_1_ generation, the duration and longevity of the spawning period, and the number of offspring produced by each F_1_ adult. During the breeding period, new-born nymphs were counted and removed from disposable cups daily. The data obtained were used to construct the age-stage two-sex life tables.

### Life table analysis

The life history data for both *S*. *graminum* and *R*. *padi* were analysed according to age-stage two-sex life table theory [39] and the method described by Chi [40]. TWOSEX-MS Chart software [41–44] was used to analyse the data and calculate the intrinsic rate of increase (*r*_*m*_), net reproductive rate (*R*_*0*_), mean generation time (*T*), finite rate of increase (*λ*) and other curve data of life table parameters. GraphPad Prism (version 3.02, GraphPad Software, San Diego, CA) was used to construct survival rate, reproductive rate, life expectancy and reproductive rate curves. Student’s t test was used to compare the developmental duration, longevity and aphid yield between the treatment and control groups, with a significance threshold of p < 0.05.

The life table parameters were calculated as follows:

Net reproductive rate:

$$R_{0}=\sum l_{X}m_{x}$$

Finite rate of increase:

$$\lambda =e^{r_{m}}$$

Intrinsic rate of increase:

$$r_{m}=\text{ln}R_{0}/T$$

Mean generation time:

$$T=\sum (xl_{x}m_{x})/\sum l_{x}m_{x}$$

In the above formula, *x* is the time interval (1 day), *l*_*x*_ is the survival rate of any individual during period *x*, and *m*_*x*_ is the average number of aphids produced per female aphid during period x.

## Results

### Lethal concentrations

The LC_50_ values of imidacloprid for *S*. *graminum* and *R*. *padi* were 3.59 and 13.78 mg L^−1^, and the LC_25_ concentrations of imidacloprid for *S*. *graminum* and *R*. *padi* were 1.13 and 7.43 mg L^−1^, respectively. *R*. *padi* was more sensitive to imidacloprid (LC_50_ = 3.59 mg L^−1^) than *S*. *graminum* (LC_50_ = 13.78 mg L^−1^) (Table 1).

**Table 1:** The lethal and sublethal concentrations of imidacloprid for *Schizaphis graminum* and *Rhopalosiphum padi*.

Species	LC_50_ (95% CI)(mg L^−1^)	LC_25_ (95% CI)(mg L^−1^)	Concentrations in treatment	Mortality (%)
*R*. *padi*	13.78 (11.70–16.41)	7.43 (5.41–9.05)	7.50	24.44
*S*. *graminum*	3.59 (2.32–1.83)	1.13 (0.38–1.83)	1.50	26.67

1) LC_50_, 50% lethal concentration; CI, confidence interval; LC_25_, 25% lethal concentration.

### Sublethal effects of imidacloprid on the longevity and fecundity of the F_0_ generation of *S*. *graminum* and *R*. *padi*

*S*. *graminum* and *R*. *padi* were treated with their respective LC_25_ of imidacloprid, and the mortality of the aphids was calculated after 24 h. The mortality rates of *S*. *graminum* and *R*. *padi* were 24.44% and 26.67%, respectively (Table 1). The surviving individuals of *S*. *graminum* and *R*. *padi* were selected and used as the F_0_ generation for subsequent life table construction. The parameter data of aphids treated with the LC_25_ of imidacloprid (the F_0_ generation) are shown in Table 2. For the F_0_ generation of *S*. *graminum* and *R*. *padi*, no significant differences were observed between the treatment and control groups in terms of longevity and fecundity (P>0.05).

**Table 2:** Sublethal effects of imidacloprid on the longevity and fecundity of the F_0_ generation of *Schizaphis graminum* and *Rhopalosiphum padi*.

Aphid species	Life table parameter	Control^1)^	Imidacloprid^2)^	t	P value
*S*. *Graminum* (Sg)	Adult longevity	11.88±0.46 a	11.33±0.38 a	0.923	0.359
	Total fecundity	21.03±0.83 a	22.73±0.76 a	-1.512	0.134
*R*. *Padi* (Rp)	Adult longevity	13.52±1.08 a	10.55±1.37 a	1.697	0.095
	Total fecundity	20.10±2.68 a	13.17±2.69 a	1.826	0.073

^1)^ Treated without insecticide, including two groups: CK_Sg_F_0_ for S. *graminum* and CK_Rp_F_0_ for *R*. *padi*.

^2)^ Treated with the sublethal dose of imidacloprid, including two groups: LC_25__Sg_F_0_ for *S*. *graminum* and LC_25__Rp_F_0_ for *R*. *padi*. Data (mean±SE) in the same row followed by different letters are significantly different (P<0.05) from those of the controls according to Student’s t test.

### Transgenerational effects on life table parameters

In the F_1_ generation of *S*. *graminum*, no significant difference was observed between the treatment and control groups in terms of the duration of the complete nymph stage (preadulthood), the duration of the adult preoviposition period (APOP), the duration of the total preoviposition period (TPOP), the reproductive period or the total longevity (P>0.05). However, the adult longevity and the total longevity of the treatment groups were significantly shorter than those of the control groups (P<0.05). In the F_1_ generation of *R*. *padi*, there were no significant differences in any life table parameters between the treatment and control groups (P<0.05) (Table 3).

**Table 3:** Sublethal effects of imidacloprid on the developmental duration and fecundity of the F_1_ generation of *Schizaphis graminum* and *Rhopalosiphum padi*.

Parameter	*S*. *graminum*			*R*. *padi*		
	CK_Sg_F_1_	LC_25__Sg_F_1_	P value	CK_Rp_F_1_	LC_25__Rp_F_1_	P value
L1 (d)	1.92±0.073	1.85±0.062	0.473	1.72±0.057	1.70±0.056	0.837
L2 (d)	1.90±0.066	1.92±0.061	0.878	1.65±0.064	1.68±0.074	0.734
L3 (d)	1.82±0.069	1.85±0.074	0.785	1.72±0.057	1.57±0.079	0.128
L4 (d)	1.95±0.061	2.03±0.064	0.453	1.68±0.051	1.58±0.90	0.337
Preadulthood (d)	7.60±0.148	7.64±0.154	0.846	6.77±0.137	6.53±0.232	0.391
APOP (d)	0.15±0.044	0.17±0.045	0.825	0.18±0.051	0.18±0.050	0.816
TPOP (d)	7.75±0.162	7.81±0.159	0.807	6.93±0.143	6.70±0.237	0.403
Oviposition period (d)	8.44±0.440	8.11±0.425	0.588	8.15±0.938	8.70±0.980	0.687
Adult longevity (d)	12.33±0.532	10.61±0.574	0.031	10.43±0.949	10.80±1.041	0.795
Total longevity (d)	20.14±0.549	18.36±0.584	0.030	17.37±0.945	17.50±1.158	0.929
Fecundity	21.92±1.248	22.28±1.320	0.843	19.50±2.559	22.67±2.716	0.400

^1)^ L1, the first nymph stage; L2, the second nymph stage; L3, the third nymph stage; L4, the fourth nymph stage; Preadulthood, the duration of the complete nymph stage; APOP, the duration of the adult preoviposition period. TPOP, the duration of the total preoviposition period.

**Fig 1:**
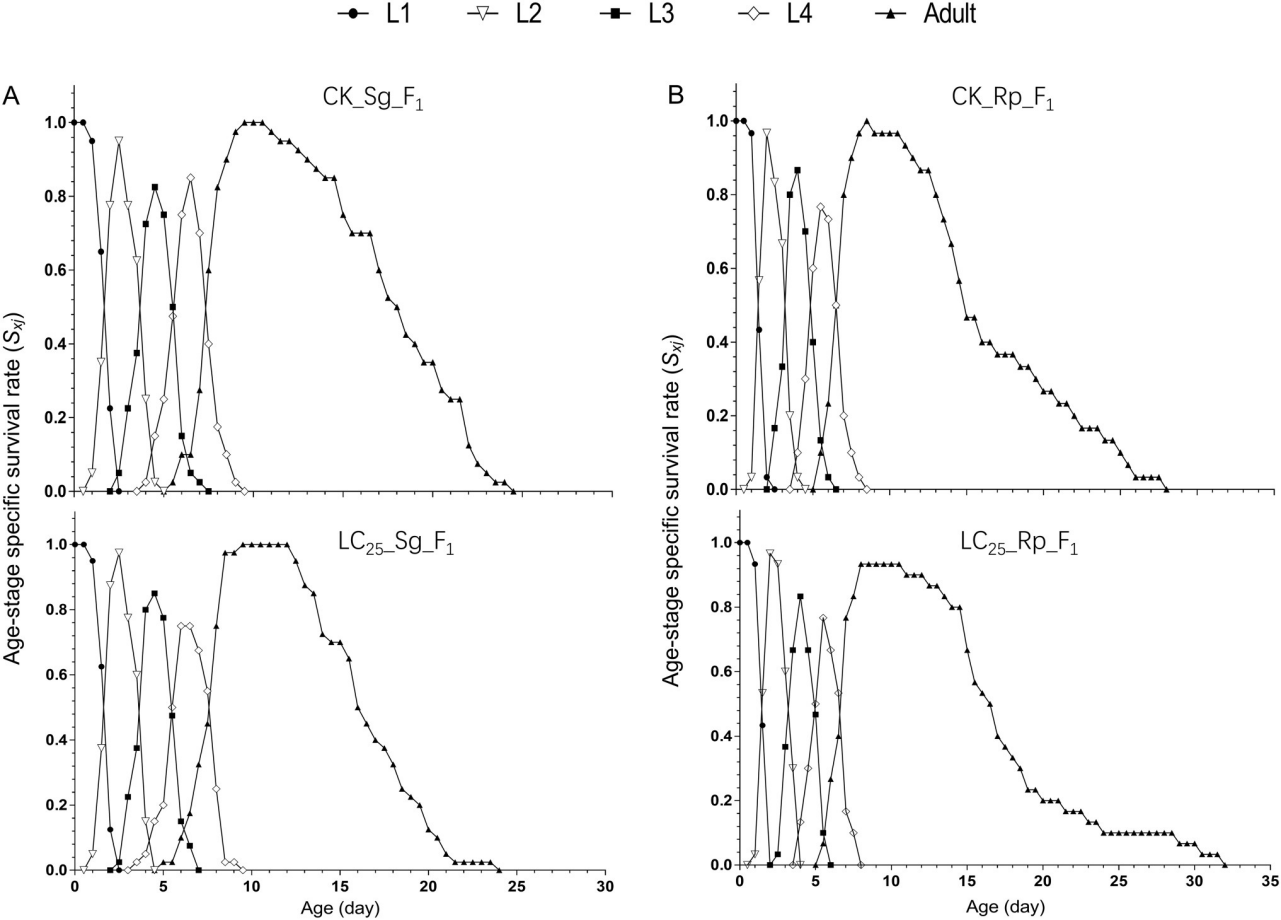
Age-stage-specific survival rates (*S*_*xj*_) of the F_1_ generation of *Schizaphis graminum* and *Rhopalosiphum padi*, with the control groups compared with the groups treated with sublethal concentrations of imidacloprid. A, *S*_*xj*_ of the F_1_ generation of *Schizaphis graminum*. B, *S*_*xj*_ of the F_1_ generation of *Rhopalosiphum padi*. L1, the first nymph stage; L2, the second nymph stage; L3, the third nymph stage; L4, the fourth nymph stage.

**Fig 2:**
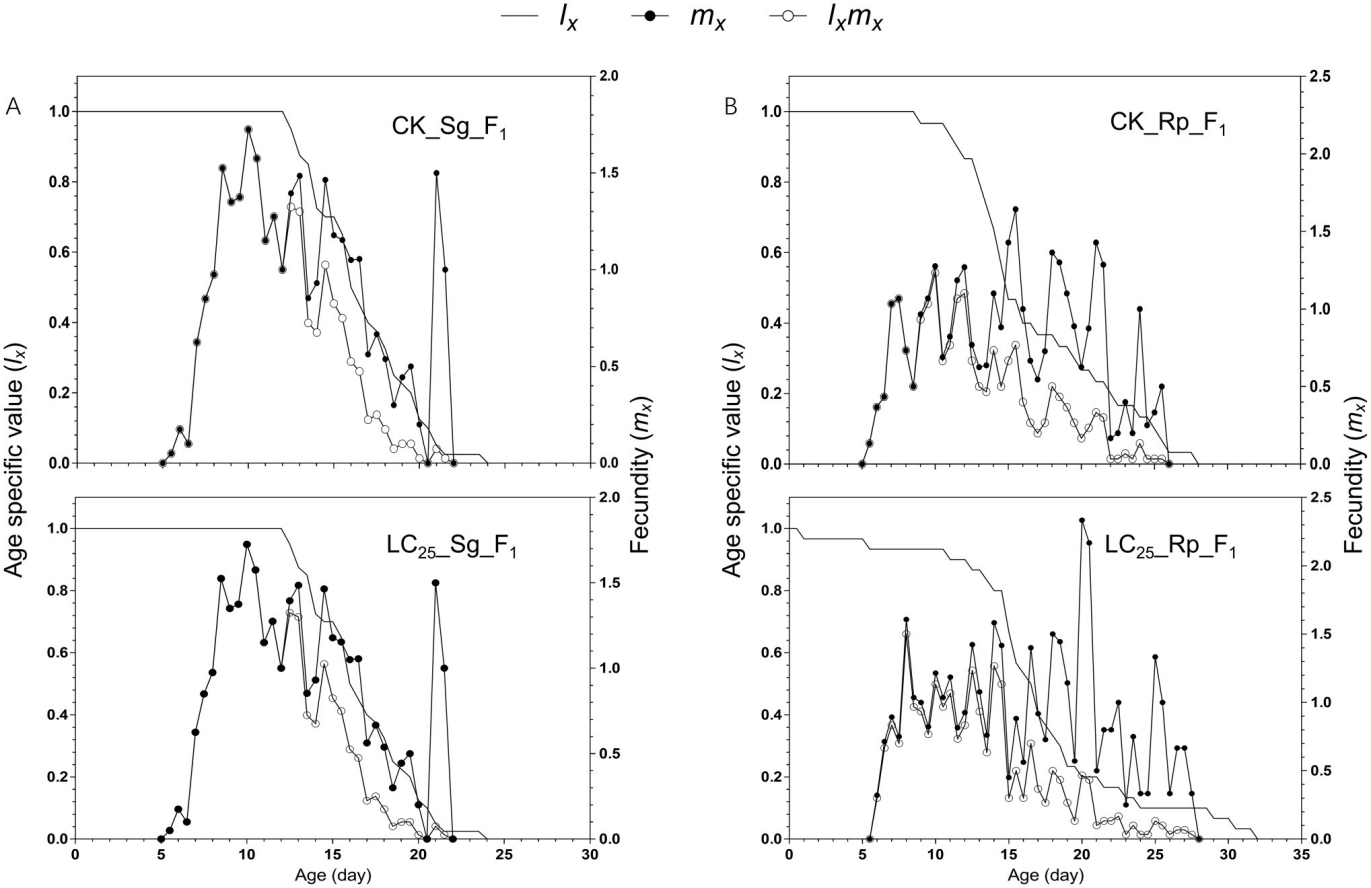
Age-specific survival rate (*l*_*x*_), age-specific fecundity of the total population (*m*_*x*_), and age-specific fertility (*l*_*x*_*m*_*x*_) of the F_1_ generation of *Schizaphis graminum* and *Rhopalosiphum padi*, with the control groups compared with the groups treated with sublethal concentrations of imidacloprid. A, *l*_*x*_, *m*_*x*_ and *l*_*x*_*m*_*x*_ of the F_1_ generation of *Schizaphis graminum*. B, *l*_*x*_, *m*_*x*_ and *l*_*x*_*m*_*x*_ of the F_1_ generation of *Rhopalosiphum padi*.

The age-stage specific survival rates (*S*_*xj*_) of both *S*. *graminum* (CK_Sg_F_1_ and LC_25__Sg_F_1_) and *R*. *padi* (CK_Rp_F_1_ and LC_25__Rp_F_1_) are shown in Fig 1A and 1B. The curve in Fig 1 reflects the probability that the new-born nymph survive to age x and period j. The survival rate curve shows the differences in survival between the CK_Sg_F_1_ group and LC_25__Sg_F_1_ group and between the CK_Rp_F_1_ group and LC_25__Rp_F_1_ group. Due to differences in developmental rates, there is significant overlap between periods (Fig 1). The age-specific reproductive rate curve (*m*_*x*_) showed that the LC_25__Sg_F_1_ group began to reproduce on the 5^th^ day, reproduction peaked on days 9 to 11, and the reproductive period ended on the 21^st^ day. In the control group (CK_Sg_F_1_), reproduction began on the 5^th^ day, peaked from days 8 to 10, and ended on the 21^st^ day (Fig 2A). The average reproductive period of the LC_25__Sg_F_1_ group was 8.11 days, similar to that of the control group (8.44 days) (Table 3). Each female aphid in the LC_25__Sg_F_1_ group produced 21.92 aphids on average, while each female aphid in the control group produced 22.28 aphids on average (Table 3). Therefore, the total fecundity of the LC_25__Sg_F_1_ group was slightly lower than that of the CK_Sg_F_1_ group, but the difference was not significant. The age-specific reproductive rate curve (m_x_) showed that the LC_25__Rp_F_1_ group began to reproduce on the 7^th^ day and stopped producing aphids on the 28^th^ day, while the control group began to reproduce on Day 5.5 and stopped producing aphids on the 26^th^ day. The fecundity of the LC_25__Rp_F_1_ and CK_Rp_F_1_ groups peaked from days 7 to 11. Females in the LC_25__Rp_F_1_ and CK_Rp_F_1_ groups produced an average of 19.5 and 22.7 aphids, respectively; this difference was not significant (Table 3). There was no evidence that imidacloprid had transgenerational effects on the age-specific survival rate (*l*_*x*_) of *S*. *graminum* and *R*. *padi* (Fig 2).

**Fig 3:**
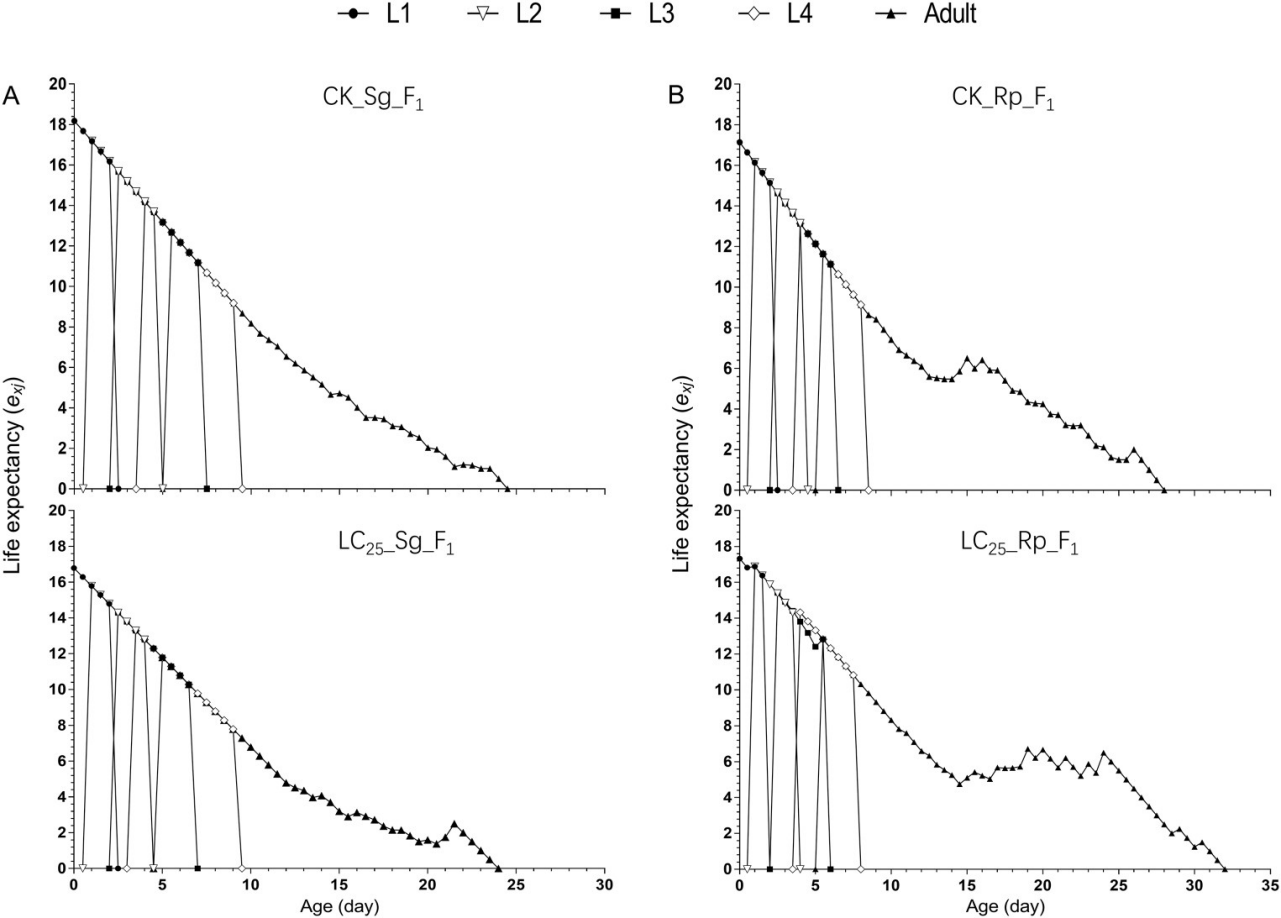
Age-stage-specific life expectancy (*e*_*xj*_) of the F_1_ generation of *Schizaphis graminum* and *Rhopalosiphum padi*, with the control groups compared with the groups treated with sublethal concentrations of imidacloprid. A, *e*_*xj*_ of the F_1_ generation of *Schizaphis graminum*. B, *e*_*xj*_ of the F_1_ generation of *Rhopalosiphum padi*.

Age-period life expectancy (*e*_*xj*_) represents the length of time that an individual aphid of age x in period j is expected to survive. The life expectancies of new-born nymphs of *S*. *graminum* in the treated and control groups were 18.5 and 17.5 days, respectively (Fig 3A). The life expectancies of new-born *R*. *padi* nymphs in the treatment and control groups were 17.3 and 17.1 days, respectively (Fig 3B).

### Transgenerational effects on population parameters

**Fig 4:**
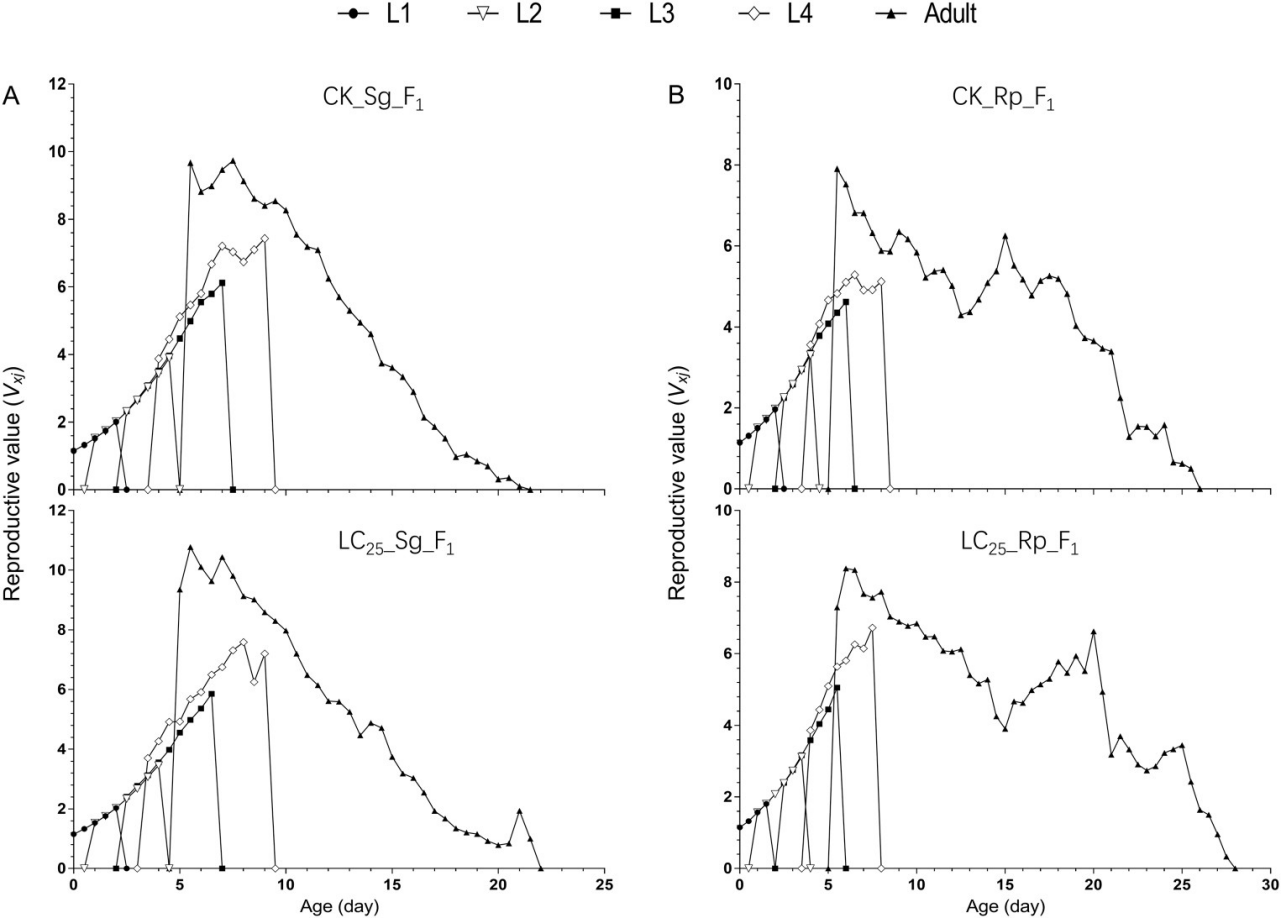
Age-stage reproductive value (*v*_*xj*_) of the F_1_ generation of *Schizaphis graminum* and *Rhopalosiphum padi*, with the control groups compared with the groups treated with sublethal concentrations of imidacloprid. A, *v*_*xj*_ of the F_1_ generation of *Schizaphis graminum*. B, *v*_*xj*_ of the F_1_ generation of *Rhopalosiphum padi*.

The LC_25_ of imidacloprid was used to treat the two wheat aphids. Subsequently, the life table parameters of the F_1_ generation were assessed and are shown in Table 4. No significant differences in life table parameters were observed between the LC_25__Sg_F_1_ and CK_Sg_F_1_ groups (of *S*. *graminum)* or between the LC_25__Rp_F_1_ and CK_Rp_F_1_ groups (of *R*. *padi)* (Table 4). The age-period reproductive rate (*V*_*xj*_) represents the expected contribution of aphid individuals at age x and period j to the future population (Fig 4). The age-period reproductive rate (*V*_*xj*_) trends of *S*. *graminum* and *R*. *padi* were similar in the treated and control groups.

**Table 4:** Sublethal effects of imidacloprid on the population parameters of the F_1_ generation of *Schizaphis graminum* and *Rhopalosiphum padi*.

Life table parameter	*S*. *graminum*		*R*. *padi*	
	CK_Sg_F_1_	LC_25__Sg_F_1_	CK_Rp_F_1_	LC_25__Rp_F_1_
Net reproductive rate	20.700±1.863 a	20.050±1.802 a	19.500±2.121 a	22.433±4.027 a
Mean generation time	22.538±0.317 a	22.284±0.713 a	21.862±1.086 a	22.010±0.553 a
Intrinsic rate of increase	0.132±0.005 a	0.134±0.007 a	0.136±0.004 a	0.136±0.006 a
Finite rate of increase	1.143±0.005 a	1.144±0.007 a	1.145±0.005 a	1.148±0.006 a

Note: Data in the same row followed by different letters are significantly different at *P* < 0.05 according to the Tukey‒Kramer test.

## Discussion

In this study, we used age-stage, two-sex life table to investigate the transgenerational sublethal effects of imidacloprid on demographic parameters of *S*. *graminum* and *R*. *padi*. Bioassay results showed that imidacloprid is high toxicity to adult *S*. *graminum* and *R*. *padi*, with LC_50_ of 3.59 and 13.78 mg L^−1^ following 24 h exposure.

Applied insecticide concentrations typically degrade to low and sublethal concentrations due to field degradation and plant growth, resulting in frequent exposure of pests to low or sublethal concentration [31]. Low or sublethal concentration of insecticides ultimately affect the physiological and behavioral traits of exposed individuals, such as lifespan, developmental period, fecundity, host finding, and feeding activity [13, 14, 45]. Therefore, indepth information about the impact of low or sublethal concentration of imidacloprid on the biological parameters might be crucial for managing *S*. *graminum* and *R*. *padi* under field contexts.

The findings demonstrated that low lethal concentration (LC_25_) of imidacloprid had no significant effects on the fecundity and longevity of directly exposed parental parental *S*. *graminum* and *R*. *padi* (F_0_). This is somewhat similar to the results that low lethal concentration of sulfoxaflor did not cause significant effects on the fecundity or the longevity of the parent generation (F_0_ generation) of either *S*. *avenae* or *R*. *padi* [46]. However, Lu et al. [47] reported that both *S*. *avenae* and *R*. *padi* exhibited significantly decreased fecundity and longevity after pulse exposure to sublethal concentrations of imidacloprid for more than three generations. Ullah et al. [48] found that LC_5_ and LC_15_ of imidacloprid significantly decreased the longevity and fecundity of melon aphids. Likewise, a short lifespan and reduced fecundity were also reported in *M*. *persicae* when exposed to sublethal concentrations of flupyradifurone [49]. These findings demonstrated that along with lethal effects, the sublethal concentrations of chemical insecticides have detrimental effects on the lifespan and fecundity of surviving aphids.

In this study, low lethal concentration of imidacloprid (LC_25_) significantly decreased the adult longevity and total longevity of progeny generation aphids (F_1_) of *S*. *graminum*, while not of *R*. *padi*. Our results are consistent with those of Vakhide and Safavi [50] that the low lethal concentrations of acetamiprid substantially decreased the longevity and fecundity of *S*. *graminum*. Furthermore, Liang et al. [51] reported the decreased longevity of *A*. *gossypii* following exposure to the LC_25_ of flupyradifurone. Similar effects have also been reported on *Sogatella furcifera* Horváth (Hemiptera: Delphacidae) and *Scolothrips longicornis* Priesner (Thysanoptera: Thripidae) when treated with low lethal concentrations of buprofezin and abamectin [52, 53]. However, some studies reported that the developmental duration of F_1_ generation insects was prolonged after the parent aphid (F_0_) was exposed to sublethal and low lethal concentrations of pesticides. Ullah et al. [54] found that the developmental duration of 1st instar *A*. *gossypii* increased when treated with LC_15_ of thiamethoxam. Likewise, Mostafiz et al. [11] reported that the low lethal concentrations of methyl benzoate extended the larval developmental time of F_1_ generation *A*. *gossypii*. This could happen when insects devote energy to the detoxification of chemical insecticides and survive at the cost of development [55, 56].

In our study, the results show that there was no significant difference in the pre-adult stage, fecundity and key demographic parameters in F_1_ individuals after exposure of parental *S*. *graminum* and *R*. *padi* (F_0_) to imidacloprid. However, it should be noted that different sublethal concentration of insecticides have different effects on the fecundity and key demographic parameters of insects. James [57] found that the recommended concentration of imidacloprid could stimulate the population growth and spawning rate of *Amblyseius victoriensis*. Cutler et al. [58] exposed *Myzus persicae* to low concentrations of imidacloprid and found that they had no obvious effects on the reproductive rate of the parents, but the reproductive rate of the offspring was changed to some extent. Koo et al. [59] reported that the LC_30_ of flonicamid significantly reduced the longevity, fecundity and net reproductive rate (R_0_) of *A*. *gossypii*. Ma et al. [12] reported that key biological parameters including *R*_*0*_, *r*, *λ*, and *F* were decreased significantly, while T and TPRP were increased when *A*. *gossypii* were exposed to the sublethal concentration of afidopyropen. Therefore, the nonlethal negative effects of chemical insecticides on the demographic parameters of individuals might affect the population growth [60].

Together, imidacloprid (LC_25_) significantly decreased the adult longevity and total longevity of progeny generation aphids (F_1_) of *S*. *graminum*, while not of *R*. *padi*. Nevertheless, LC_25_ of imidacloprid had no significant effects on the fecundity and longevity of directly exposed parental parental *S*. *graminum* and *R*. *padi* (F_0_). Besides, there was no significant difference in the pre-adult stage, fecundity and key demographic parameters in F_1_ individuals after exposure of parental *S*. *graminum* and *R*. *padi* (F_0_) to imidacloprid. These results provides indepth information about the overall effects of imidacloprid on *S*. *graminum* and *R*. *padi* that might help to manage these two key pests.

## Supporting information

S1 File (ZIP)
