# Supplementary material for: Sublethal effects of imidacloprid on the fitness of two species of wheat aphids, Schizaphis graminum (R.) and Rhopalosiphum padi (L.)
Source: PLoS One. 2023 Nov 27;18(11):e0294877. doi: 10.1371/journal.pone.0294877 (PMC10681248; doi:10.1371/journal.pone.0294877)
Supplement: S1 File — (ZIP) [file pone.0294877.s001.zip › 毕设数据/禾谷缢管蚜(Rhopalosiphum padi Linnaeus)/123.doc]

ONEWAY 成蚜寿命 产蚜量 BY 处理组
  /POLYNOMIAL=1
  /STATISTICS DESCRIPTIVES HOMOGENEITY
  /PLOT MEANS
  /MISSING ANALYSIS
  /POSTHOC=BTUKEY LSD T2 T3 WALLER(100) ALPHA(0.05).


单向


附注	
创建的输出	24-4月-2022 20时49分21秒	
注释	 	
输入	活动的数据集	数据集0	
	过滤器	<none>	
	权重	<none>	
	拆分文件	<none>	
	工作数据文件中的 N 行	90	
缺失值处理	缺失定义	用户定义的缺失值以缺失对待。	
	使用的案例	每个分析的统计量都基于对于该分析中的任意变量都没有缺失数据的案例。	
语法	ONEWAY 成蚜寿命 产蚜量 BY 处理组
  /POLYNOMIAL=1
  /STATISTICS DESCRIPTIVES HOMOGENEITY
  /PLOT MEANS
  /MISSING ANALYSIS
  /POSTHOC=BTUKEY LSD T2 T3 WALLER(100) ALPHA(0.05).
	
资源	处理器时间	00 00:00:01.469	
	已用时间	00 00:00:01.060	


[数据集0] 


描述	
	N	均值	标准差	标准误	均值的 95% 置信区间	
					下限	上限	
成蚜寿命	CK	30	13.52	5.915	1.080	11.31	15.73	
	LC25	30	10.55	7.527	1.374	7.74	13.36	
	LC50	30	8.42	7.235	1.321	5.72	11.12	
	总数	90	10.83	7.165	.755	9.33	12.33	
产蚜量	CK	30	20.10	14.681	2.680	14.62	25.58	
	LC25	30	13.17	14.730	2.689	7.67	18.67	
	LC50	30	8.07	7.506	1.370	5.26	10.87	
	总数	90	13.78	13.560	1.429	10.94	16.62	

描述	
	极小值	极大值	
成蚜寿命	CK	5	25	
	LC25	0	24	
	LC50	0	25	
	总数	0	25	
产蚜量	CK	3	53	
	LC25	0	53	
	LC50	0	23	
	总数	0	53	


方差齐性检验	
	Levene 统计量	df1	df2	显著性	
成蚜寿命	.994	2	87	.374	
产蚜量	3.163	2	87	.047	


ANOVA	
	平方和	df	均方	F	显著性	
成蚜寿命	组间	（组合）	393.622	2	196.811	4.100	.020	
		线性项	对比	390.150	1	390.150	8.128	.005	
			偏差	3.472	1	3.472	.072	.789	
	组内	4175.958	87	48.000			
	总数	4569.581	89				
产蚜量	组间	（组合）	2188.822	2	1094.411	6.716	.002	
		线性项	对比	2172.017	1	2172.017	13.329	.000	
			偏差	16.806	1	16.806	.103	.749	
	组内	14176.733	87	162.951			
	总数	16365.556	89				


在此之后检验


多重比较	
因变量	(I) 处理组	(J) 处理组	均值差 (I-J)	标准误	显著性	
成蚜寿命	LSD	CK	LC25	2.967	1.789	.101	
			LC50	5.100*	1.789	.005	
		LC25	CK	-2.967	1.789	.101	
			LC50	2.133	1.789	.236	
		LC50	CK	-5.100*	1.789	.005	
			LC25	-2.133	1.789	.236	
	Tamhane	CK	LC25	2.967	1.748	.260	
			LC50	5.100*	1.706	.012	
		LC25	CK	-2.967	1.748	.260	
			LC50	2.133	1.906	.607	
		LC50	CK	-5.100*	1.706	.012	
			LC25	-2.133	1.906	.607	
	Dunnett T3	CK	LC25	2.967	1.748	.257	
			LC50	5.100*	1.706	.012	
		LC25	CK	-2.967	1.748	.257	
			LC50	2.133	1.906	.603	
		LC50	CK	-5.100*	1.706	.012	
			LC25	-2.133	1.906	.603	
产蚜量	LSD	CK	LC25	6.933*	3.296	.038	
			LC50	12.033*	3.296	.000	
		LC25	CK	-6.933*	3.296	.038	
			LC50	5.100	3.296	.125	
		LC50	CK	-12.033*	3.296	.000	
			LC25	-5.100	3.296	.125	
	Tamhane	CK	LC25	6.933	3.797	.203	
			LC50	12.033*	3.010	.001	
		LC25	CK	-6.933	3.797	.203	
			LC50	5.100	3.018	.267	
		LC50	CK	-12.033*	3.010	.001	
			LC25	-5.100	3.018	.267	
	Dunnett T3	CK	LC25	6.933	3.797	.201	
			LC50	12.033*	3.010	.001	
		LC25	CK	-6.933	3.797	.201	
			LC50	5.100	3.018	.263	
		LC50	CK	-12.033*	3.010	.001	
			LC25	-5.100	3.018	.263	

多重比较	
因变量	(I) 处理组	(J) 处理组	95% 置信区间	
			下限	上限	
成蚜寿命	LSD	CK	LC25	-.59	6.52	
			LC50	1.54	8.66	
		LC25	CK	-6.52	.59	
			LC50	-1.42	5.69	
		LC50	CK	-8.66	-1.54	
			LC25	-5.69	1.42	
	Tamhane	CK	LC25	-1.34	7.27	
			LC50	.90	9.30	
		LC25	CK	-7.27	1.34	
			LC50	-2.55	6.82	
		LC50	CK	-9.30	-.90	
			LC25	-6.82	2.55	
	Dunnett T3	CK	LC25	-1.33	7.27	
			LC50	.90	9.30	
		LC25	CK	-7.27	1.33	
			LC50	-2.55	6.81	
		LC50	CK	-9.30	-.90	
			LC25	-6.81	2.55	
产蚜量	LSD	CK	LC25	.38	13.48	
			LC50	5.48	18.58	
		LC25	CK	-13.48	-.38	
			LC50	-1.45	11.65	
		LC50	CK	-18.58	-5.48	
			LC25	-11.65	1.45	
	Tamhane	CK	LC25	-2.40	16.27	
			LC50	4.56	19.51	
		LC25	CK	-16.27	2.40	
			LC50	-2.40	12.60	
		LC50	CK	-19.51	-4.56	
			LC25	-12.60	2.40	
	Dunnett T3	CK	LC25	-2.39	16.26	
			LC50	4.57	19.50	
		LC25	CK	-16.26	2.39	
			LC50	-2.39	12.59	
		LC50	CK	-19.50	-4.57	
			LC25	-12.59	2.39	

	
*. 均值差的显著性水平为 0.05。
	


同类子集


成蚜寿命	
	处理组	N	alpha = 0.05 的子集	
			1	2	
Tukey Ba	LC50	30	8.42		
	LC25	30	10.55	10.55	
	CK	30		13.52	
Waller-Duncana,b	LC50	30	8.42		
	LC25	30	10.55	10.55	
	CK	30		13.52	
将显示同类子集中的组均值。	
a. 将使用调和均值样本大小 = 30.000。
b. 类型 1/类型 2 错误严重性比值 = 100。
	


产蚜量	
	处理组	N	alpha = 0.05 的子集	
			1	2	
Tukey Ba	LC50	30	8.07		
	LC25	30	13.17	13.17	
	CK	30		20.10	
Waller-Duncana,b	LC50	30	8.07		
	LC25	30	13.17	13.17	
	CK	30		20.10	
将显示同类子集中的组均值。	
a. 将使用调和均值样本大小 = 30.000。
b. 类型 1/类型 2 错误严重性比值 = 100。
	


均值图
