# Supplementary material for: Sublethal effects of imidacloprid on the fitness of two species of wheat aphids, Schizaphis graminum (R.) and Rhopalosiphum padi (L.)
Source: PLoS One. 2023 Nov 27;18(11):e0294877. doi: 10.1371/journal.pone.0294877 (PMC10681248; doi:10.1371/journal.pone.0294877)
Supplement: S1 File — (ZIP) [file pone.0294877.s001.zip › 毕设数据/禾谷缢管蚜(Rhopalosiphum padi Linnaeus)/456.doc]

ONEWAY 一龄若虫龄期 二龄若虫龄期 三龄若虫龄期 四龄若虫龄期 若蚜历期 世代历期 生殖期 成蚜寿命 全世代 产蚜量 BY 处理
  /POLYNOMIAL=1
  /STATISTICS DESCRIPTIVES HOMOGENEITY
  /PLOT MEANS
  /MISSING ANALYSIS
  /POSTHOC=BTUKEY LSD T2 T3 WALLER(100) ALPHA(0.05).


单向


附注	
创建的输出	24-4月-2022 20时37分12秒	
注释	 	
输入	活动的数据集	数据集0	
	过滤器	<none>	
	权重	<none>	
	拆分文件	<none>	
	工作数据文件中的 N 行	90	
缺失值处理	缺失定义	用户定义的缺失值以缺失对待。	
	使用的案例	每个分析的统计量都基于对于该分析中的任意变量都没有缺失数据的案例。	
语法	ONEWAY 一龄若虫龄期 二龄若虫龄期 三龄若虫龄期 四龄若虫龄期 若蚜历期 世代历期 生殖期 成蚜寿命 全世代 产蚜量 BY 处理
  /POLYNOMIAL=1
  /STATISTICS DESCRIPTIVES HOMOGENEITY
  /PLOT MEANS
  /MISSING ANALYSIS
  /POSTHOC=BTUKEY LSD T2 T3 WALLER(100) ALPHA(0.05).
	
资源	处理器时间	00 00:00:03.500	
	已用时间	00 00:00:02.797	


[数据集0] 


描述	
	N	均值	标准差	标准误	均值的 95% 置信区间	
					下限	上限	
一龄若虫龄期	CK	30	1.72	.313	.057	1.60	1.83	
	LC25	30	1.70	.311	.057	1.58	1.82	
	LC50	30	1.72	.313	.057	1.60	1.83	
	总数	90	1.71	.309	.033	1.65	1.78	
二龄若虫龄期	CK	30	1.65	.351	.064	1.52	1.78	
	LC25	30	1.68	.404	.074	1.53	1.83	
	LC50	30	1.73	.286	.052	1.63	1.84	
	总数	90	1.69	.348	.037	1.62	1.76	
三龄若虫龄期	CK	30	1.72	.313	.057	1.60	1.83	
	LC25	30	1.57	.430	.079	1.41	1.73	
	LC50	30	1.48	.594	.108	1.26	1.71	
	总数	90	1.59	.466	.049	1.49	1.69	
四龄若虫龄期	CK	30	1.68	.278	.051	1.58	1.79	
	LC25	30	1.58	.493	.090	1.40	1.77	
	LC50	30	1.35	.671	.123	1.10	1.60	
	总数	90	1.54	.521	.055	1.43	1.65	
若蚜历期	CK	30	6.77	.751	.137	6.49	7.05	
	LC25	30	6.53	1.273	.232	6.06	7.01	
	LC50	30	6.28	1.271	.232	5.81	6.76	
	总数	90	6.53	1.130	.119	6.29	6.76	
世代历期	CK	30	6.93	.785	.143	6.64	7.23	
	LC25	30	6.70	1.297	.237	6.22	7.18	
	LC50	30	6.40	1.342	.245	5.90	6.90	
	总数	90	6.68	1.176	.124	6.43	6.92	
生殖期	CK	30	8.15	5.135	.937	6.23	10.07	
	LC25	30	8.70	5.368	.980	6.70	10.70	
	LC50	30	7.55	4.793	.875	5.76	9.34	
	总数	90	8.13	5.069	.534	7.07	9.19	
成蚜寿命	CK	30	10.43	5.196	.949	8.49	12.37	
	LC25	30	10.80	5.700	1.041	8.67	12.93	
	LC50	30	10.15	6.267	1.144	7.81	12.49	
	总数	90	10.46	5.679	.599	9.27	11.65	
全世代	CK	30	17.37	5.178	.945	15.43	19.30	
	LC25	30	17.50	6.341	1.158	15.13	19.87	
	LC50	30	16.55	7.258	1.325	13.84	19.26	
	总数	90	17.14	6.259	.660	15.83	18.45	
产蚜量	CK	30	19.50	14.017	2.559	14.27	24.73	
	LC25	30	22.67	14.877	2.716	17.11	28.22	
	LC50	30	23.07	16.787	3.065	16.80	29.33	
	总数	90	21.74	15.183	1.600	18.56	24.92	

描述	
	极小值	极大值	
一龄若虫龄期	CK	1	3	
	LC25	1	2	
	LC50	1	2	
	总数	1	3	
二龄若虫龄期	CK	1	2	
	LC25	0	2	
	LC50	1	2	
	总数	0	2	
三龄若虫龄期	CK	1	2	
	LC25	0	2	
	LC50	0	2	
	总数	0	2	
四龄若虫龄期	CK	2	3	
	LC25	0	2	
	LC50	0	2	
	总数	0	3	
若蚜历期	CK	6	9	
	LC25	1	8	
	LC50	3	8	
	总数	1	9	
世代历期	CK	6	9	
	LC25	1	8	
	LC50	3	8	
	总数	1	9	
生殖期	CK	1	20	
	LC25	0	21	
	LC50	0	17	
	总数	0	21	
成蚜寿命	CK	2	23	
	LC25	0	25	
	LC50	0	23	
	总数	0	25	
全世代	CK	10	29	
	LC25	1	32	
	LC50	3	30	
	总数	1	32	
产蚜量	CK	2	53	
	LC25	0	56	
	LC50	0	61	
	总数	0	61	


方差齐性检验	
	Levene 统计量	df1	df2	显著性	
一龄若虫龄期	.010	2	87	.990	
二龄若虫龄期	.307	2	87	.736	
三龄若虫龄期	1.127	2	87	.329	
四龄若虫龄期	4.360	2	87	.016	
若蚜历期	1.385	2	87	.256	
世代历期	1.702	2	87	.188	
生殖期	.096	2	87	.909	
成蚜寿命	.420	2	87	.659	
全世代	1.156	2	87	.320	
产蚜量	.643	2	87	.528	


ANOVA	
	平方和	df	均方	F	显著性	
一龄若虫龄期	组间	（组合）	.006	2	.003	.028	.972	
		线性项	对比	.000	1	.000	.000	1.000	
			偏差	.006	1	.006	.057	.812	
	组内	8.483	87	.098			
	总数	8.489	89				
二龄若虫龄期	组间	（组合）	.106	2	.053	.430	.652	
		线性项	对比	.104	1	.104	.848	.360	
			偏差	.001	1	.001	.011	.916	
	组内	10.683	87	.123			
	总数	10.789	89				
三龄若虫龄期	组间	（组合）	.839	2	.419	1.978	.145	
		线性项	对比	.817	1	.817	3.851	.053	
			偏差	.022	1	.022	.105	.747	
	组内	18.450	87	.212			
	总数	19.289	89				
四龄若虫龄期	组间	（组合）	1.756	2	.878	3.416	.037	
		线性项	对比	1.667	1	1.667	6.485	.013	
			偏差	.089	1	.089	.346	.558	
	组内	22.358	87	.257			
	总数	24.114	89				
若蚜历期	组间	（组合）	3.506	2	1.753	1.384	.256	
		线性项	对比	3.504	1	3.504	2.767	.100	
			偏差	.001	1	.001	.001	.974	
	组内	110.175	87	1.266			
	总数	113.681	89				
世代历期	组间	（组合）	4.289	2	2.144	1.570	.214	
		线性项	对比	4.267	1	4.267	3.123	.081	
			偏差	.022	1	.022	.016	.899	
	组内	118.867	87	1.366			
	总数	123.156	89				
生殖期	组间	（组合）	19.850	2	9.925	.381	.684	
		线性项	对比	5.400	1	5.400	.207	.650	
			偏差	14.450	1	14.450	.555	.458	
	组内	2266.550	87	26.052			
	总数	2286.400	89				
成蚜寿命	组间	（组合）	6.372	2	3.186	.097	.908	
		线性项	对比	1.204	1	1.204	.037	.849	
			偏差	5.168	1	5.168	.157	.693	
	组内	2864.242	87	32.922			
	总数	2870.614	89				
全世代	组间	（组合）	15.872	2	7.936	.199	.820	
		线性项	对比	10.004	1	10.004	.251	.618	
			偏差	5.868	1	5.868	.147	.702	
	组内	3471.142	87	39.898			
	总数	3487.014	89				
产蚜量	组间	（组合）	229.089	2	114.544	.491	.614	
		线性项	对比	190.817	1	190.817	.818	.368	
			偏差	38.272	1	38.272	.164	.686	
	组内	20288.033	87	233.196			
	总数	20517.122	89				


在此之后检验


多重比较	
因变量	(I) 处理	(J) 处理	均值差 (I-J)	标准误	显著性	95% 置信区间	
						下限	上限	
一龄若虫龄期	LSD	CK	LC25	.017	.081	.837	-.14	.18	
			LC50	.000	.081	1.000	-.16	.16	
		LC25	CK	-.017	.081	.837	-.18	.14	
			LC50	-.017	.081	.837	-.18	.14	
		LC50	CK	.000	.081	1.000	-.16	.16	
			LC25	.017	.081	.837	-.14	.18	
	Tamhane	CK	LC25	.017	.081	.996	-.18	.21	
			LC50	.000	.081	1.000	-.20	.20	
		LC25	CK	-.017	.081	.996	-.21	.18	
			LC50	-.017	.081	.996	-.21	.18	
		LC50	CK	.000	.081	1.000	-.20	.20	
			LC25	.017	.081	.996	-.18	.21	
	Dunnett T3	CK	LC25	.017	.081	.996	-.18	.21	
			LC50	.000	.081	1.000	-.20	.20	
		LC25	CK	-.017	.081	.996	-.21	.18	
			LC50	-.017	.081	.996	-.21	.18	
		LC50	CK	.000	.081	1.000	-.20	.20	
			LC25	.017	.081	.996	-.18	.21	
二龄若虫龄期	LSD	CK	LC25	-.033	.090	.713	-.21	.15	
			LC50	-.083	.090	.360	-.26	.10	
		LC25	CK	.033	.090	.713	-.15	.21	
			LC50	-.050	.090	.582	-.23	.13	
		LC50	CK	.083	.090	.360	-.10	.26	
			LC25	.050	.090	.582	-.13	.23	
	Tamhane	CK	LC25	-.033	.098	.981	-.27	.21	
			LC50	-.083	.083	.682	-.29	.12	
		LC25	CK	.033	.098	.981	-.21	.27	
			LC50	-.050	.090	.927	-.27	.17	
		LC50	CK	.083	.083	.682	-.12	.29	
			LC25	.050	.090	.927	-.17	.27	
	Dunnett T3	CK	LC25	-.033	.098	.981	-.27	.21	
			LC50	-.083	.083	.678	-.29	.12	
		LC25	CK	.033	.098	.981	-.21	.27	
			LC50	-.050	.090	.926	-.27	.17	
		LC50	CK	.083	.083	.678	-.12	.29	
			LC25	.050	.090	.926	-.17	.27	
三龄若虫龄期	LSD	CK	LC25	.150	.119	.210	-.09	.39	
			LC50	.233	.119	.053	.00	.47	
		LC25	CK	-.150	.119	.210	-.39	.09	
			LC50	.083	.119	.485	-.15	.32	
		LC50	CK	-.233	.119	.053	-.47	.00	
			LC25	-.083	.119	.485	-.32	.15	
	Tamhane	CK	LC25	.150	.097	.338	-.09	.39	
			LC50	.233	.123	.179	-.07	.54	
		LC25	CK	-.150	.097	.338	-.39	.09	
			LC50	.083	.134	.900	-.25	.41	
		LC50	CK	-.233	.123	.179	-.54	.07	
			LC25	-.083	.134	.900	-.41	.25	
	Dunnett T3	CK	LC25	.150	.097	.334	-.09	.39	
			LC50	.233	.123	.177	-.07	.54	
		LC25	CK	-.150	.097	.334	-.39	.09	
			LC50	.083	.134	.898	-.25	.41	
		LC50	CK	-.233	.123	.177	-.54	.07	
			LC25	-.083	.134	.898	-.41	.25	
四龄若虫龄期	LSD	CK	LC25	.100	.131	.447	-.16	.36	
			LC50	.333*	.131	.013	.07	.59	
		LC25	CK	-.100	.131	.447	-.36	.16	
			LC50	.233	.131	.078	-.03	.49	
		LC50	CK	-.333*	.131	.013	-.59	-.07	
			LC25	-.233	.131	.078	-.49	.03	
	Tamhane	CK	LC25	.100	.103	.710	-.16	.36	
			LC50	.333*	.133	.048	.00	.66	
		LC25	CK	-.100	.103	.710	-.36	.16	
			LC50	.233	.152	.343	-.14	.61	
		LC50	CK	-.333*	.133	.048	-.66	.00	
			LC25	-.233	.152	.343	-.61	.14	
	Dunnett T3	CK	LC25	.100	.103	.705	-.16	.36	
			LC50	.333*	.133	.048	.00	.66	
		LC25	CK	-.100	.103	.705	-.36	.16	
			LC50	.233	.152	.340	-.14	.61	
		LC50	CK	-.333*	.133	.048	-.66	.00	
			LC25	-.233	.152	.340	-.61	.14	
若蚜历期	LSD	CK	LC25	.233	.291	.424	-.34	.81	
			LC50	.483	.291	.100	-.09	1.06	
		LC25	CK	-.233	.291	.424	-.81	.34	
			LC50	.250	.291	.392	-.33	.83	
		LC50	CK	-.483	.291	.100	-1.06	.09	
			LC25	-.250	.291	.392	-.83	.33	
	Tamhane	CK	LC25	.233	.270	.775	-.43	.90	
			LC50	.483	.270	.220	-.18	1.15	
		LC25	CK	-.233	.270	.775	-.90	.43	
			LC50	.250	.328	.833	-.56	1.06	
		LC50	CK	-.483	.270	.220	-1.15	.18	
			LC25	-.250	.328	.833	-1.06	.56	
	Dunnett T3	CK	LC25	.233	.270	.770	-.43	.90	
			LC50	.483	.270	.217	-.18	1.15	
		LC25	CK	-.233	.270	.770	-.90	.43	
			LC50	.250	.328	.830	-.56	1.06	
		LC50	CK	-.483	.270	.217	-1.15	.18	
			LC25	-.250	.328	.830	-1.06	.56	
世代历期	LSD	CK	LC25	.233	.302	.442	-.37	.83	
			LC50	.533	.302	.081	-.07	1.13	
		LC25	CK	-.233	.302	.442	-.83	.37	
			LC50	.300	.302	.323	-.30	.90	
		LC50	CK	-.533	.302	.081	-1.13	.07	
			LC25	-.300	.302	.323	-.90	.30	
	Tamhane	CK	LC25	.233	.277	.788	-.45	.92	
			LC50	.533	.284	.186	-.17	1.24	
		LC25	CK	-.233	.277	.788	-.92	.45	
			LC50	.300	.341	.764	-.54	1.14	
		LC50	CK	-.533	.284	.186	-1.24	.17	
			LC25	-.300	.341	.764	-1.14	.54	
	Dunnett T3	CK	LC25	.233	.277	.784	-.45	.92	
			LC50	.533	.284	.184	-.17	1.24	
		LC25	CK	-.233	.277	.784	-.92	.45	
			LC50	.300	.341	.761	-.54	1.14	
		LC50	CK	-.533	.284	.184	-1.24	.17	
			LC25	-.300	.341	.761	-1.14	.54	
生殖期	LSD	CK	LC25	-.550	1.318	.677	-3.17	2.07	
			LC50	.600	1.318	.650	-2.02	3.22	
		LC25	CK	.550	1.318	.677	-2.07	3.17	
			LC50	1.150	1.318	.385	-1.47	3.77	
		LC50	CK	-.600	1.318	.650	-3.22	2.02	
			LC25	-1.150	1.318	.385	-3.77	1.47	
	Tamhane	CK	LC25	-.550	1.356	.969	-3.88	2.78	
			LC50	.600	1.282	.954	-2.55	3.75	
		LC25	CK	.550	1.356	.969	-2.78	3.88	
			LC50	1.150	1.314	.768	-2.08	4.38	
		LC50	CK	-.600	1.282	.954	-3.75	2.55	
			LC25	-1.150	1.314	.768	-4.38	2.08	
	Dunnett T3	CK	LC25	-.550	1.356	.968	-3.88	2.78	
			LC50	.600	1.282	.953	-2.55	3.75	
		LC25	CK	.550	1.356	.968	-2.78	3.88	
			LC50	1.150	1.314	.764	-2.08	4.38	
		LC50	CK	-.600	1.282	.953	-3.75	2.55	
			LC25	-1.150	1.314	.764	-4.38	2.08	
成蚜寿命	LSD	CK	LC25	-.367	1.481	.805	-3.31	2.58	
			LC50	.283	1.481	.849	-2.66	3.23	
		LC25	CK	.367	1.481	.805	-2.58	3.31	
			LC50	.650	1.481	.662	-2.29	3.59	
		LC50	CK	-.283	1.481	.849	-3.23	2.66	
			LC25	-.650	1.481	.662	-3.59	2.29	
	Tamhane	CK	LC25	-.367	1.408	.991	-3.83	3.10	
			LC50	.283	1.486	.997	-3.37	3.94	
		LC25	CK	.367	1.408	.991	-3.10	3.83	
			LC50	.650	1.547	.966	-3.15	4.45	
		LC50	CK	-.283	1.486	.997	-3.94	3.37	
			LC25	-.650	1.547	.966	-4.45	3.15	
	Dunnett T3	CK	LC25	-.367	1.408	.991	-3.83	3.09	
			LC50	.283	1.486	.997	-3.37	3.94	
		LC25	CK	.367	1.408	.991	-3.09	3.83	
			LC50	.650	1.547	.965	-3.15	4.45	
		LC50	CK	-.283	1.486	.997	-3.94	3.37	
			LC25	-.650	1.547	.965	-4.45	3.15	
全世代	LSD	CK	LC25	-.133	1.631	.935	-3.37	3.11	
			LC50	.817	1.631	.618	-2.42	4.06	
		LC25	CK	.133	1.631	.935	-3.11	3.37	
			LC50	.950	1.631	.562	-2.29	4.19	
		LC50	CK	-.817	1.631	.618	-4.06	2.42	
			LC25	-.950	1.631	.562	-4.19	2.29	
	Tamhane	CK	LC25	-.133	1.495	1.000	-3.81	3.55	
			LC50	.817	1.628	.944	-3.20	4.83	
		LC25	CK	.133	1.495	1.000	-3.55	3.81	
			LC50	.950	1.760	.932	-3.38	5.28	
		LC50	CK	-.817	1.628	.944	-4.83	3.20	
			LC25	-.950	1.760	.932	-5.28	3.38	
	Dunnett T3	CK	LC25	-.133	1.495	1.000	-3.81	3.54	
			LC50	.817	1.628	.943	-3.19	4.83	
		LC25	CK	.133	1.495	1.000	-3.54	3.81	
			LC50	.950	1.760	.930	-3.37	5.27	
		LC50	CK	-.817	1.628	.943	-4.83	3.19	
			LC25	-.950	1.760	.930	-5.27	3.37	
产蚜量	LSD	CK	LC25	-3.167	3.943	.424	-11.00	4.67	
			LC50	-3.567	3.943	.368	-11.40	4.27	
		LC25	CK	3.167	3.943	.424	-4.67	11.00	
			LC50	-.400	3.943	.919	-8.24	7.44	
		LC50	CK	3.567	3.943	.368	-4.27	11.40	
			LC25	.400	3.943	.919	-7.44	8.24	
	Tamhane	CK	LC25	-3.167	3.732	.784	-12.34	6.01	
			LC50	-3.567	3.993	.756	-13.39	6.26	
		LC25	CK	3.167	3.732	.784	-6.01	12.34	
			LC50	-.400	4.095	1.000	-10.47	9.67	
		LC50	CK	3.567	3.993	.756	-6.26	13.39	
			LC25	.400	4.095	1.000	-9.67	10.47	
	Dunnett T3	CK	LC25	-3.167	3.732	.780	-12.33	6.00	
			LC50	-3.567	3.993	.753	-13.38	6.25	
		LC25	CK	3.167	3.732	.780	-6.00	12.33	
			LC50	-.400	4.095	1.000	-10.46	9.66	
		LC50	CK	3.567	3.993	.753	-6.25	13.38	
			LC25	.400	4.095	1.000	-9.66	10.46	
*. 均值差的显著性水平为 0.05。
	


同类子集


一龄若虫龄期	
	处理	N	alpha = 0.05 的子集	
			1	
Tukey Ba	LC25	30	1.70	
	CK	30	1.72	
	LC50	30	1.72	
将显示同类子集中的组均值。	
a. 将使用调和均值样本大小 = 30.000。
	


二龄若虫龄期	
	处理	N	alpha = 0.05 的子集	
			1	
Tukey Ba	CK	30	1.65	
	LC25	30	1.68	
	LC50	30	1.73	
将显示同类子集中的组均值。	
a. 将使用调和均值样本大小 = 30.000。
	


三龄若虫龄期	
	处理	N	alpha = 0.05 的子集	
			1	
Tukey Ba	LC50	30	1.48	
	LC25	30	1.57	
	CK	30	1.72	
将显示同类子集中的组均值。	
a. 将使用调和均值样本大小 = 30.000。
	


四龄若虫龄期	
	处理	N	alpha = 0.05 的子集	
			1	2	
Tukey Ba	LC50	30	1.35		
	LC25	30	1.58	1.58	
	CK	30		1.68	
Waller-Duncana,b	LC50	30	1.35		
	LC25	30	1.58	1.58	
	CK	30		1.68	
将显示同类子集中的组均值。	
a. 将使用调和均值样本大小 = 30.000。
b. 类型 1/类型 2 错误严重性比值 = 100。
	


若蚜历期	
	处理	N	alpha = 0.05 的子集	
			1	
Tukey Ba	LC50	30	6.28	
	LC25	30	6.53	
	CK	30	6.77	
将显示同类子集中的组均值。	
a. 将使用调和均值样本大小 = 30.000。
	


世代历期	
	处理	N	alpha = 0.05 的子集	
			1	
Tukey Ba	LC50	30	6.40	
	LC25	30	6.70	
	CK	30	6.93	
将显示同类子集中的组均值。	
a. 将使用调和均值样本大小 = 30.000。
	


生殖期	
	处理	N	alpha = 0.05 的子集	
			1	
Tukey Ba	LC50	30	7.55	
	CK	30	8.15	
	LC25	30	8.70	
将显示同类子集中的组均值。	
a. 将使用调和均值样本大小 = 30.000。
	


成蚜寿命	
	处理	N	alpha = 0.05 的子集	
			1	
Tukey Ba	LC50	30	10.15	
	CK	30	10.43	
	LC25	30	10.80	
将显示同类子集中的组均值。	
a. 将使用调和均值样本大小 = 30.000。
	


全世代	
	处理	N	alpha = 0.05 的子集	
			1	
Tukey Ba	LC50	30	16.55	
	CK	30	17.37	
	LC25	30	17.50	
将显示同类子集中的组均值。	
a. 将使用调和均值样本大小 = 30.000。
	


产蚜量	
	处理	N	alpha = 0.05 的子集	
			1	
Tukey Ba	CK	30	19.50	
	LC25	30	22.67	
	LC50	30	23.07	
将显示同类子集中的组均值。	
a. 将使用调和均值样本大小 = 30.000。
	


均值图
