# Supplementary material for: Sublethal effects of imidacloprid on the fitness of two species of wheat aphids, Schizaphis graminum (R.) and Rhopalosiphum padi (L.)
Source: PLoS One. 2023 Nov 27;18(11):e0294877. doi: 10.1371/journal.pone.0294877 (PMC10681248; doi:10.1371/journal.pone.0294877)
Supplement: S1 File — (ZIP) [file pone.0294877.s001.zip › 毕设数据/禾谷缢管蚜(Rhopalosiphum padi Linnaeus)/文本原始数据.docx]

CK

1,F,3,4,3,5,15

1,2,0,2,1,0,1,1,2,0,1,1,0,0,-1

2,F,2,3,4,4,32

1,1,0,0,1,2,2,1,0,0,2,1,1,2,3,3,0,0,3,3,0,0,1,2,2,2,0,0,1,1,1,1,-1

3,F,3,3,4,4,37

0,1,1,0,1,1,2,1,0,1,3,1,2,0,0,2,1,0,0,0,0,2,2,0,0,2,3,-1

4,F,4,4,3,3,15

2,1,0,0,3,2,1,0,3,3,2,4,-1

5,F,5,4,4,4,10

0,1,1,1,1,0,-1

6,F,4,4,4,3,37

1,1,0,0,0,0,2,1,0,0,3,2,2,2,0,0,3,3,2,2,0,0,2,4,0,0,0,2,0,0,1,0,-1

7,F,3,2,4,3,17

2,1,0,0,2,1,0,0,1,2,-1

8,F,3,3,3,3,18

1,0,0,2,2,0,5,3,0,0,1,1,3,1,0,0,3,2,-1

9,F,3,3,2,3,45

0,2,1,0,0,0,1,1,2,0,0,3,2,0,0,1,2,3,1,2,3,0,1,0,0,4,4,1,2,2,3,0,0,0,0,2,1,3,1,1,1,-1

10,F,3,3,3,4,35

0,2,3,0,0,1,1,0,0,3,0,3,0,3,2,2,2,3,3,2,1,1,1,2,2,1,2,0,0,4,4,0,0,-1

11,F,4,3,3,3,15

0,2,1,0,0,2,0,3,0,-1

12,F,3,4,4,3,14

3,2,0,0,0,0,4,3,-1

13,F,4,3,3,3,13

0,1,1,0,0,0,0,2,1,0,0,1,0,-1

14,F,3,4,4,3,20

1,1,0,0,0,1,-1

15,F,4,4,2,3,16

2,3,0,2,1,0,0,1,2,1,0,2,3,1,-1

16,F,3,4,4,3,33

3,2,0,0,0,0,4,1,0,0,2,1,0,0,3,0,6,1,0,1,0,0,,0,0,3,2,0,0,2,2,-1

17,F,4,3,4,3,36

0,3,2,0,0,2,3,0,0,7,4,3,0,0,2,1,3,1,2,0,3,4,3,3,0,0,0,3,1,2,0,0,0,0,1,-1

18,F,,4,4,4,4,14

2,1,1,2,1,0,0,2,3,1,0,0,-1

19,F,3,2,3,3,31

3,2,0,1,1,2,0,0,2,1,0,0,0,2,1,0,0,1,2,1,1,1,2,0,0,2,-1

20,F,4,3,3,4,25

3,0,0,2,1,1,1,0,2,2,0,0,0,2,0,0,1,3,-1

21,F,3,4,4,3,4

0,3,0,-1

22,F,3,2,3,3,13

1,2,2,2,3,0,0,2,0,2,2,-1

23,F,4,3,3,3,19

1,0,1,0,3,0,0,2,0,0,1,1,0,1,-1

24,F,3,4,3,3,13

4,3,2,2,0,0,1,2,-1

25,F,4,3,3,3,24

0,1,1,0,0,0,0,2,3,0,0,2,0,1,1,3,5,0,0,1,1,-1

26,F,4,4,4,4,24

2,3,0,0,2,0,5,6,0,0,1,1,0,0,3,5,-1

27,F,3,4,4,3,23

1,0,2,1,2,2,0,1,-1

28,F,3,2,4,3,20

0,0,2,1,0,1,0,2,1,0,4,4,2,1,-1

29,F,3,3,3,3,11

2,1,0,0,0,0,3,5,-1

30,F,4,3,4,4,17

1,0,0,1,-1

25

1,F,4,4,4,4,17

2,2,0,0,2,0,0,1,0,0,3,2,2,2,-1

2,F,3,3,4,4,8

1,1,3,1,-1

3,F,3,3,2,3,26

0,2,2,0,1,0,1,0,0,1,4,0,2,0,0,2,2,4,2,-1

4,F,4,3,3,3,19

1,0,0,2,1,0,6,2,0,0,1,0,0,0,3,2,-1

5,F,3,4,4,3,21

0,1,0,0,2,0,1,2,2,0,0,3,3,0,1,0,0,2,0,6,2,2,2,-1

6,F,4,3,4,4,49

3,2,0,2,0,1,0,1,0,0,1,4,0,1,0,0,2,0,3,1,2,3,2,2,1,3,1,1,2,0,2,0,1,0,1,0,0,0,1,1,-1

7,F,3,3,3,3,15

1,0,0,0,3,2,-1

8,N,2,-1

9,F,4,4,3,3,20

1,0,6,2,1,1,4,4,0,0,3,2,0,0,1,2,4,1,1,0,-1

10,F,3,4,4,3,17

0,1,1,0,0,0,0,1,0,1,1,-1

11,F,2,3,3,4,18

1,1,0,0,0,1,0,0,3,3,0,0,1,1,0,0,4,5,-1

12,F,3,4,3,3,15

2,1,2,2,1,1,-1

13,F,3,4,4,3,44

2,2,0,0,3,2,0,0,5,3,0,0,0,1,1,2,0,0,0,3,1,2,1,2,0,0,2,3,0,1,0,0,1,0,0,0,4,3,1,1,1,1,-1

14,F,4,3,4,4,33

0,2,1,0,0,2,2,0,0,3,2,0,0,2,1,4,1,0,0,1,0,3,1,0,1,2,1,1,1,-1

15,F,3,3,4,4,11

4,2,3,1,2,2,-1

16,F,3,3,2,4,34

1,0,1,1,3,3,0,0,2,1,0,0,4,2,0,0,1,1,0,0,1,1,2,1,4,2,0,0,4,5,-1

17,F,3,3,2,3,32

0,2,1,0,0,3,1,0,2,0,0,4,2,0,2,0,0,3,7,0,0,3,6,1,1,3,5,2,2,2,3,0,1,-1

18,F,4,3,3,3,25

5,2,0,0,2,1,2,2,0,0,1,0,1,1,0,2,0,0,3,1,-1-

19,F,4,4,4,4,45

3,4,0,0,2,3,2,1,4,8,0,0,3,4,0,0,3,1,0,0,1,1,2,0,1,0,0,0,4,3,0,2,1-1

20,F,3,3,3,4,25

0,2,1,0,0,1,0,1,0,2,1,3,7,0,0,2,2,1,6,0,0,1,1,-1

21,F,3,3,3,3,22

0,5,3,2,1,0,0,2,0,1,0,1,1,2,0,4,2,-1-

22,F,4,4,3,3,22

2,0,0,0,3,1,2,2,2,0,1,1,0,0,1,-1

23,F,4,4,3,3,17

2,0,1,0,2,1,0,0,3,1,2,0,2,1,0,1,-1

24,F,4,4,3,3,16

0,0,3,2,0,0,3,1,2,1,0,2,0,0,1,0,3,2,1,1,-1-

25,F,3,4,3,4,20

2,2,0,0,1,0,3,2,2,1,0,0,3,2,-1-

26,F,3,3,3,3,19

2,0,0,0,2,1,0,0,1,1,3,2,0,1,-1

27,N,4,4,3,-1

28,F,4,4,4,4,14

2,1,2,2,3,0,-1

29,F,3,3,3,3,18

0,3,0,1,1,2,1,0,0,2,2,0,0,0,1,7,2,2,1,-1

30,F,4,4,3,3,26

2,1,0,0,3,1,0,0,4,2,2,2,3,0,3,3,0,0,0,1,2,1,0,0,2,-1

50

1,F,3,4,3,4,1-

0,1,1,0,0,2,1,0,0,1,1,0,0,3,-1

2,N,4,3,2,-1

3,F,2,4,3,4,27

3,2,2,2,0,0,2,0,0,0,4,3,2,5,0,0,2,1,-1-

4,F,4,3,4,4,26

1,1,0,0,1,0,2,3,0,0,2,1,3,0,5,5,0,0,1,1,3,1,-1-

5,F,4,3,4,4,26

2,0,3,1,0,0,2,0,3,1,0,0,3,1,0,0,2,2,-1

6,N,4,4,-1

7,N,3,4,2,-1

8,F,3,4,3,4,28

1,0,0,0,3,2,3,3,0,0,2,2,2,1,0,0,5,5,2,1,0,0,2,2,-1

9,F,3,4,4,3,27

0,2,1,3,2,0,0,2,2,2,1,1,1,0,0,2,2,3,1,0,0,3,4,-1

10,F,3,3,3,3,12

2,1,2,0,0,0,1,0,0,2,1,2,-1

11,F,3,3,3,3,46

2,3,0,0,2,2,0,0,5,3,0,0,2,1,1,2,1,1,4,2,0,1,0,0,3,2,3,3,2,2,1,0,2,0,-1

12,N,3,4,-1

13,F,3,3,4,3,29

1,1,0,0,1,1,3,1,2,1,5,6,2,3,4,4,0,0,2,2,1,1,-1

14,F,3,3,4,3,27

4,3,0,0,2,1,2,2,6,6,0,0,4,2,2,2,0,0,1,3,-1

15,F,4,4,3,3,46

5,6,0,0,2,2,1,2,0,0,6,5,1,1,0,0,2,1,3,2,1,1,3,5,0,0,2,3,2,2,0,1,1,1,-1

16,F,4,3,2,3,13

0,1,1,0,0,3,1,2,1,3,2,-1

17,N,3,2,-1

18,F,4,4,3,2,25

3,2,0,0,2,2,3,1,0,0,0,1,-1

19,F,4,4,3,3,22

2,2,0,0,3,0,0,0,1,3,2,2,0,0,0,1,3,4,3,5,-1

20,F,4,4,3,3,39

0,1,0,0,3,2,2,2,0,0,1,1,4,4,0,0,1,1,3,5,2,3,3,4,1,2,2,7,1,2,0,1,-1

21,F,4,4,3,3,18

3,1,1,1,2,3,4,5,-1

22,F,4,4,4,4,28

3,2,1,2,1,2,0,0,2,0,3,2,0,0,5,6,-1

23,F,3,3,4,4,26

0,0,2,1,0,0,4,4,0,0,2,3,0,1,3,1,0,0,0,1,0,0,2,2,2,2,-1

24,F,3,3,4,3,24

4,6,0,0,1,3,5,5,0,0,6,7,-1

25,F,3,4,3,4,22

2,0,0,1,0,2,0,0,1,3,2,2,3,4,-1

26,F,4,3,4,3,11

2,0,1,1,3,2,0,0,1,2,4,-1

27,F,4,3,4,3,17

1,1,2,1,0,2,1,1,-1

28,F,4,3,4,2,23

0,1,0,0,0,1,1,0,1,2,1,0,0,3,2,0,1,0,0,2,2,-1

29,F,4,4,3,3,22

1,0,0,0,1,1,0,0,2,2,3,3,2,2,0,0,2,4,-1

30,F,2,3,3,3,12

2,2,2,1,0,3,0,1,1,1,2,-1
